# Rare Cases of Polymyalgia Rheumatica After Receiving COVID-19 Vaccinations

**DOI:** 10.7759/cureus.37782

**Published:** 2023-04-18

**Authors:** Todd Furr, Manisha Garg

**Affiliations:** 1 Internal Medicine, Ascension Providence Hospital, Southfield, USA

**Keywords:** pro-inflammatory cytokines, autoinflammatory syndrome from adjuvants, covid-19, vaccinations, polymyalgia rheumatica

## Abstract

Polymyalgia rheumatica (PMR) is a systemic rheumatic inflammatory disease of adults presenting with symmetrical proximal muscle stiffness and pain predominantly involving the shoulders, neck, and pelvic girdle. The coronavirus disease of 2019 (COVID-19) presented as a pandemic causing worldwide morbidity and mortality in large numbers. Rapid scientific research expedited preventative vaccine development and has helped tremendously in cutting down severe illness, hospitalizations, and death from COVID-19, with the messenger ribonucleic acid (mRNA) vaccines outperforming the others. We present two cases that showcase the incidence of polymyalgia rheumatica after receiving COVID-19 vaccination. Patient 1 is a 69-year-old female who developed arm and thigh stiffness a week before the second dose while receiving her primary Moderna vaccine series. She had an elevated C-reactive protein (CRP) and erythrocyte sedimentation rate (ESR), so she was started on low-dose steroids, which were weaned down over a five-month period. Three weeks after receiving her Moderna booster, she had a recurrence of the classic polymyalgia rheumatica symptoms and elevated ESR. She responded to prednisone 15 mg with a successful taper over eight months. Patient 2 is a 74-year-old male who received his primary series and booster through Pfizer-BioNTech. Prior to the booster, he was treated for COVID-19 with monoclonal antibody therapy. He presented to the office with hip and shoulder pain and stiffness along with an elevated C-reactive protein. Consequently, he received 20 mg of prednisone but needed to increase his dose to 25 mg total to help with the control of his inflammation.

The goal of this article is to prompt physicians about the possibility of PMR incidence after patients receive vaccinations for severe acute respiratory syndrome coronavirus 2 (SARS-CoV-2). PMR can be debilitating to the quality of life of patients. Knowing this association allows for more timely and competent treatment. PMR following SARS-CoV-2 vaccinations is continuously being observed in the medical field. Increased knowledge may help prevent the recurrence with subsequent doses. Further studies on the follow-up of such cases and the effect on subsequent immunization would be helpful.

## Introduction

Polymyalgia rheumatica (PMR) is a common disorder in patients over 50 years of age presenting with pain in proximal joints associated with morning stiffness along with elevated inflammatory markers erythrocyte sedimentation rate (ESR) and/or C-reactive protein (CRP). It is the second most common rheumatic condition in people who are older than 50 years of age after rheumatoid arthritis [1]. The incidence rate seems to peak in the eighth decade of life and is more common in females than in males [1]. This can cause distress for patients and make ordinary activities more difficult. Treatment for these patients includes low-dose steroids responding within 4-6 weeks. Steroids such as prednisone have shown great outcomes and improvement in symptoms.

A common association with PMR is the incidence of giant cell arteritis (GCA). The common clinical presentation for this condition includes headaches, acute vision loss, and jaw claudication [2]. Treatment for this condition includes high-dose steroids. Investigating these symptoms is important for clinicians to not miss because doing so can lead to permanent blindness for the patient.

Severe acute respiratory syndrome coronavirus 2 (SARS-CoV-2) first emerged in Wuhan, China, in 2019, which started a global healthcare and financial crisis. With the widespread and quick nature of the virus, the healthcare community developed vaccinations in a timely manner. Approved vaccinations include mRNA (Pfizer-BioNTech and Moderna), protein subunit vaccine (Novavax), and viral vector vaccines (J&J/Janssen + Oxford-AstraZeneca). These vaccinations prime the immune system by providing memory to promote efficiency and quickness with exposure [3]. Common side effects include pain at the injection site, allergic skin reactions, flu-like symptoms, headache, fatigue, and fever [3]. These manifestations are different for every patient. Minor adverse effects being more common, SARS-COV-2 vaccinations have shown other disease manifestations post-vaccination with no clear understanding of why.

## Case presentation

We present two cases that show the incidence of polymyalgia rheumatica after administering vaccines for COVID-19. The characteristics of the disease are variable for both patients, and a summary of both patients is found in Table 1.

**Table 1:** Patient characteristics, vaccine demographics, laboratory values, and treatment CRP: C-reactive protein, ESR: erythrocyte sedimentation rate

Patient number	Age/sex	Painful sites	Vaccine provider	Dose	CRP level (mg/L)	ESR level (mm/hour)	Initial treatment	Outcome
1	69/female	Shoulders, pelvic girdle	Moderna	Second dose of primary series, first booster	31.1 mg/L	68 mm/hour, 57 mm/hour	Prednisone 20 mg total, prednisone 15 mg total	Faster improvement
2	74/male	Shoulders, pelvic girdle, and neck	Pfizer-BioNTech	First booster	73.2 mg/L	24 mm/hour	Prednisone 20 mg total, prednisone 25 mg total	Slower improvement

Patient 1 is a 69-year-old Caucasian female who presented in April 2021 to the office with hip and severe bilateral shoulder pain. Her vital signs were unremarkable. She received her primary COVID-19 series through Moderna in March and April 2021. During the visit, she denied cough, dyspnea, jaw claudication, or vision changes. This ruled out any temporal arteritis or other inflammatory causes.

The patient received an injection of Depo-Medrol 40 mg/mL to help her with the pain until her laboratory work came back. The laboratory findings showed an erythrocyte sedimentation rate of 68 mm/hour and a C-reactive protein of 31.1 mg/L. However, other antibodies were negative, including antinuclear antibody (ANA), creatine phosphokinase (CK), rheumatoid factor (RF), and anti-cyclic citrulline pep IgG. She was prescribed prednisone 20 mg and was weaned off over a five-month period. She responded well to low-dose steroids, eradicating her symptoms.

She received her Moderna booster in November 2021. During that visit, she did not have any symptoms of PMR and was stable with her other chronic conditions including arthritis, anxiety, cervical spondylosis, depression, osteoporosis, and vitamin B12 deficiency (non-anemic). She returned to the office in January 2022 in distress stating that her muscle weakness and pain have returned. Her ESR was ordered and came back high at 57 mm/hour. Consequently, she was commenced on prednisone 15 mg with a successful taper over the next eight months.

Patient 2 is a 74-year-old Caucasian male who received his primary series through Pfizer-BioNTech in April and May 2021. He also received a monoclonal antibody infusion for COVID-19 in November 2021. After these vaccinations and infusion, he did not have any symptoms of polymyalgia rheumatica. He received his booster from Pfizer-BioNTech in March 2022, just 20 days before the onset of his symptoms.

The symptoms he encountered included excruciating pain in his bilateral hips, shoulder, and posterior neck. He had difficulty lifting his arms up and getting out of his chair. He denied that he had any unexpected weight loss, night sweats, nasal congestion, cough, sore throat, nausea, vomiting, headache, blurred vision, or jaw claudication.

He was given depo-medrol 40 mg/mL suspension for his pain. Laboratory values that were ordered included deoxyribonucleic acid (DNA) double-stranded antibody, ANA, CK, RF, and anti-cyclic citrulline pep IgG. All these laboratory values were negative. His C-reactive protein came back at 73.2 mg/L; therefore, he was commenced on prednisone 10 mg tablets twice daily. However, he did not fully recover; thus, the prednisone dose was increased to 25 mg divided to help control his inflammation. He is currently still receiving this treatment.

## Discussion

As COVID-19 continues to affect people worldwide, it is important to notice inflammatory events that may arise from SARS-CoV-2 vaccinations. Knowing that a disease such as polymyalgia rheumatica may occur afterward may put hesitance with physicians giving subsequent boosters.

Our main hypothesis stems from a condition known as autoinflammatory/inflammatory syndrome from adjuvants (ASIA). This is considered when there is a cluster or related immune-mediated diseases that develop in an individual after being exposed to an adjuvant [4,5]. We mainly want to focus our attention on toll-like receptor 7 (TLR-7) and toll-like receptor 9 (TLR-9) nucleic acid receptors that are found in the mRNA vaccine. There is a debate that these have an increased expression in PMR etiology [6]. With the activation of TLR-7 and TLR-9 in COVID-19 mRNA vaccines, there are more alterations with pro-inflammatory cytokines such as interleukin 1 (IL-1) and interleukin 6 (IL-6). Other alterations that have been discussed include changes in T helper cell type 1 (TH1) and T helper cell type 17 (TH17) balance and a decline in dehydroepiandrosterone or androstenedione when exposed to a particular adjuvant [7,8]. The symptoms that are seen with this syndrome include myalgias, arthritis, neuronal damage, fatigue, encephalitis, and vasculitis, some of which were seen in our patients.

Inflammatory cytokines lead to the activation of innate and adaptive immunity causing inflammatory conditions. Innate inflammatory disorders include GCA, PMR, and Crohn’s disease, while adaptive immunity is undifferentiated connective tissue disease and Sjogren’s syndrome. This could be a possibility as the role of vaccinations is to prime the immune system for future exposure.

Other explanations are unclear as some believe that infection may have an implication as PMR incidence occurs more frequently during the winter and during epidemics of mycoplasma, chlamydia pneumonia, and parvovirus B19 infections [7]. With our patients, these etiologies did not seem to manifest as their symptoms were not evident in one particular season. The timing of the disease course after the SARS-CoV-2 vaccination suggests otherwise, and more data needs to be received to say it is a cause-effect relationship.

Incidences of inflammatory conditions have been discussed in the literature following different vaccinations. Although rare, literature does show cases where inflammatory conditions arise from influenza vaccinations [9-11]. This should be noted when discussing the different etiologies that rise after COVID-19 vaccinations. Different diseases have been reported around the world, including subacute thyroiditis and Graves’ disease, after receiving Pfizer-BioNTech [12,13]. Others include inflammatory arthritis, Behcet’s disease, and systemic lupus erythematosus [14]. Overall, these incidences are very uncommon, and the link is uncertain, just like our two patients. The literature is sparse in describing an answer to this dilemma, and this may cause many physicians to seek out risk-benefit analysis with delivering primary series or booster doses.

## Conclusions

In conclusion, the literature does not have the answers to why inflammatory conditions such as polymyalgia rheumatica occur after receiving SARS-CoV-2 vaccinations. With the possibility of other viral outbreaks and the need for immunizations, physicians need to be more aware of these conditions to help prevent exacerbation from the primary series and boosters. With both case presentations, there is hesitation to give future boosters to these individuals due to the impact on their quality of life. This can be a transient or reversible phenomenon, but more research needs to be conducted in the preface of inflammatory mediators increasing after being exposed to an adjuvant. This can help physicians seek out risk-benefit analysis in COVID-19 booster immunizations, aiding in improving the quality of life of patients.
